# One Year’s Treatment with the Glucagon-Like Peptide 1 Receptor Agonist Liraglutide Decreases Hepatic Fat Content in Women with Nonalcoholic Fatty Liver Disease and Prior Gestational Diabetes Mellitus in a Randomized, Placebo-Controlled Trial

**DOI:** 10.3390/jcm9103213

**Published:** 2020-10-06

**Authors:** Louise Vedtofte, Emilie Bahne, Signe Foghsgaard, Jonatan I. Bagger, Camilla Andreasen, Charlotte Strandberg, Peter M. Gørtz, Jens J. Holst, Henning Grønbæk, Jens A. Svare, Tine D. Clausen, Elisabeth R. Mathiesen, Peter Damm, Lise L. Gluud, Filip K. Knop, Tina Vilsbøll

**Affiliations:** 1 Steno Diabetes Center Copenhagen, Niels Steensens Vej 2, 2820 Gentofte, Denmark; louise.vedtofte@regionh.dk (L.V.); emilie_bahne@hotmail.com (E.B.); signe.foghsgaard@regionh.dk (S.F.); jonatan.ising.bagger@regionh.dk (J.I.B.); cam.andreasen@gmail.com (C.A.); filip.krag.knop.01@regionh.dk (F.K.K.); 2 Center for Clinical Metabolic Research, Gentofte Hospital, University of Copenhagen, Gentofte Hospitalsvej 7, 3rd floor, 2900 Hellerup, Denmark; 3 Danish Diabetes Academy, Odense University Hospital, Kløvervænget 6, Entrance 93, 8th floor, 5000 Odense C, Denmark; 4 Department of Radiology, Gentofte Hospital, University of Copenhagen, Gentofte Hospitalsvej 4A, 2900 Hellerup, Denmark; charlotte.strandberg@regionh.dk; 5 Department of Nuclear Medicine, Gentofte Hospital, University of Copenhagen, Gentofte Hospitalsvej 2, 1st floor, 2900 Hellerup, Denmark; peter.michael.goertz.02@regionh.dk; 6 Department of Biomedical Sciences, Faculty of Health and Medical Sciences, University of Copenhagen, Blegdamsvej 3B, 2200 Copenhagen N, Denmark; jjholst@sund.ku.dk; 7 Novo Nordisk Foundation Center for Basic Metabolic Research, Faculty of Health and Medical Sciences, University of Copenhagen, Blegdamsvej 3B, 2200 Copenhagen N, Denmark; 8 Department of Hepatology & Gastroenterology, Aarhus University Hospital, University of Aarhus, Palle Juul-Jensens Boulevard 99, 8200 Aarhus N, Denmark; henning.gronbaek@aarhus.rm.dk; 9 Department of Obstetrics and Gynaecology, Herlev Hospital, University of Copenhagen, Borgmester Ib Juuls Vej 21, 2730 Herlev, Denmark; jens.svare@dadlnet.dk; 10 Department of Gynaecology and Obstetrics, Nordsjaellands Hospital, University of Copenhagen, Dyrehavevej 29, 3400 Hillerød, Denmark; tine.clausen@regionh.dk; 11 Center for Pregnant Women with Diabetes, Department of Endocrinology, Rigshospitalet, University of Copenhagen, Blegdamsvej 9, 2100 Copenhagen Ø, Denmark; elisabeth.reinhardt.mathiesen@regionh.dk; 12 Department of Clinical Medicine, Faculty of Health and Medical Sciences, University of Copenhagen, Blegdamsvej 3B, 2200 Copenhagen N, Denmark; nis.peter.damm@regionh.dk (P.D.); lise.lotte.gluud.01@regionh.dk (L.L.G.); 13 Center for Pregnant Women with Diabetes, Department of Obstetrics, Rigshospitalet, University of Copenhagen, Blegdamsvej 9, 2100 Copenhagen Ø, Denmark; 14 Gastrounit, Hvidovre Hospital, University of Copenhagen, Kettegård Allé 30, 2650 Hvidovre, Denmark

**Keywords:** gestational diabetes mellitus, fatty liver, GLP-1 analogue, GLP-1 receptor agonist, hepatic fat, liraglutide, NAFLD

## Abstract

Prior gestational diabetes mellitus (pGDM) is associated with increased risk of nonalcoholic fatty liver disease (NAFLD). Treatment with glucagon-like peptide 1 (GLP-1) receptor agonists has shown beneficial effects in NAFLD patients. We evaluated the effect of the GLP-1 analogue liraglutide on NAFLD features in women with pGDM. Eighty-two overweight/obese, nondiabetic women with pGDM were included. We performed abdominal ultrasound, transient elastography with controlled attenuation parameter (CAP), and blood sampling at baseline and after 1 year. Thirty-seven women were randomized to liraglutide (1.8 mg once-daily) and 45 to placebo. Based on the ultrasound scan, 18 women (22%) had ultrasound-verified NAFLD at baseline and of these, 10 (56%) received liraglutide treatment. After 1 year, eight participants no longer had steatosis, four in each treatment group. The number of participants who developed NAFLD was similar in the two treatment groups; five in the liraglutide group and six in the placebo group (*p* = 0.74). Compared to placebo, liraglutide reduced the CAP-assessed intrahepatic fat content (−28 (−44;−11) vs. 2 (−13;18) dB/m, *p <* 0.01) and body weight (−4.7 (−6.4;−2.9) vs. −1.4 (−3;0.3) kg, *p <* 0.01). One-year’s liraglutide treatment had no effect on the presence of ultrasound-diagnosed NAFLD in overweight/obese nondiabetic women with pGDM, but reduced body weight and steatosis assessed by transient elastography with CAP.

## 1. Introduction

Obesity, type 2 diabetes, dyslipidemia, and insulin resistance are closely associated with nonalcoholic fatty liver disease (NAFLD) [1,2]. NAFLD is defined by the presence of steatosis in >5% of hepatocytes based on histological analysis or by a fat fraction >5.6% assessed by proton magnetic resonance spectroscopy or quantitative fat/water selective magnetic resonance imaging after the exclusion of secondary causes of fat accumulation in the liver [3]. NAFLD covers a spectrum of conditions ranging from simple steatosis over nonalcoholic steatohepatitis (NASH), which may lead to fibrosis and cirrhosis [3]. NAFLD is currently the most common chronic liver disease in the Western world [4] with a prevalence of 20–33% among adults [5] and up to 70% in adults with type 2 diabetes [6]. NAFLD is strongly associated with cardiovascular disease, which is the leading cause of death in patients with NAFLD and NASH with fibrosis [7]. Presently, biomarkers including aspartate aminotransferase (AST), alanine aminotransferase (ALT), gamma-glutamyltransferase (GGT), soluble CD163 (sCD163), are being used for noninvasive monitoring of hepatic inflammation, injury, and recovery [8,9].

Gestational diabetes mellitus (GDM) is defined as glucose intolerance recognized during pregnancy, and in Europe, Australia, and North America it affects 2–9% of pregnant women. We recently showed a high prevalence of NAFLD in relatively young and nonseverely obese women with prior GDM (pGDM) [10]. Several studies have indicated beneficial effects of glucagon-like peptide 1 (GLP-1) receptor agonists (GLP-1RA) on NAFLD and NASH [11,12,13,14,15,16,17,18,19,20,21,22,23,24,25]. Liraglutide is a GLP-1RA used for the treatment of type 2 diabetes and obesity. Recently, liraglutide was shown to reduce the risk of major cardiovascular events in patients with type 2 diabetes and established or high risk of cardiovascular disease [21]. In addition, liraglutide has been shown to reduce inflammation in diet-induced obese rats with NAFLD [19] as well as in patients with overweight and type 2 diabetes [12] and patients with NAFLD with/without type 2 diabetes [15,26].

We hypothesized that 1 year’s treatment with liraglutide (1.8 mg s.c. once daily) would prevent the development of NAFLD or resolve NAFLD in a population of overweight or obese women with pGDM but without current diabetes. Exploratively, we also examined if the effect of liraglutide was different in pGDM women with and without NAFLD, since this has not previously been investigated in any population.

## 2. Materials and Methods

### 2.1. Study Design and Outcomes

This paper describes the analyses of a prespecified secondary endpoint of an investigator-initiated, randomized, placebo-controlled, double-blinded intervention trial carried out during 1 year in nondiabetic women with pGDM [10]. The primary endpoint of the study was the change in glucose tolerance from baseline to week 52 as measured by the AUC for the plasma glucose excursion following a 4 h 75 g-OGTT. The objective of this analysis was to evaluate the proportion of women with ultrasound-verified NAFLD in a cohort of women with pGDM after 1 year’s treatment with liraglutide (1.8 mg once-daily) vs. placebo. The two abdominal ultrasound scans took place before the first study dose and 1 to 14 days after the last study dose. Additional objectives included estimation of change from baseline to year one in circulating liver enzymes, inflammatory markers, cholesterols, glucagon, and steatosis measured by transient elastography; controlled attenuation parameter (CAP) as measured by the FibroScan^®^; fatty liver index (FLI), whole body and visceral fat mass, and android-to-gynoid fat mass ratio measured by dual energy X-ray absorptiometry (DXA) scan. 

### 2.2. Participants and Recruitment

One hundred and five nondiabetic women with pGDM were recruited in the main study of whom one was excluded due to prior GLP-1RA use. All women were diagnosed with GDM according to the cutoff in the Danish guideline, which is a plasma glucose ≥9.0 mmol/L 2 h after a 75 g-OGTT. Inclusion and exclusion criteria have been published previously including the extension of the time since last GDM pregnancy from 5 to 10 years, due to slow recruitment rate [27]. Participants were recruited from august 2012 to august 2014 and were followed-up for 1 year after their randomization to the study. Of the 104 participants, 100 women attended B-mode ultrasonographic evaluation of the liver for presence or absence of steatosis. Baseline details of the included participants have previously been published [10]. Fifteen women discontinued in the trial of whom nine were in the placebo group. The reasons for discontinuation in the placebo group were lack of completion of experimental days (1), personal reasons (5), deep venous thrombosis (1), and lost to follow-up (2), and in the liraglutide group, the causes were unwillingness to take study drug (1), pregnancy (1), ulcerative colitis requiring steroid treatment (1), personal reasons (1), and lost to follow-up (2). In addition, three women did not attend their 1-year ultrasound scan, leaving 82 women with pGDM for the present analyses of whom 45 received placebo treatment and 37 received liraglutide. The protocol was approved by the Danish Medicines Agency (EudraCT number: 2012-001371-27) and the Scientific-Ethical Committee of the Capital Region of Denmark (H-2-2012-073), registered with the Danish Data Protection Agency (01714 GEH-2012-024) and at clinicaltrialsregister.eu (EudraCT number: 2012-001371-37), carried out under surveillance and guidance of the Good Clinical Practice (GCP) unit at Copenhagen University Hospital, Copenhagen, Denmark, in compliance with the International Conference on Harmonisation GCP guideline and conducted in accordance with the Helsinki Declaration. All authors had access to the study data and reviewed and approved the final manuscript.

### 2.3. Procedures

The experimental procedures had previously been described in detail [10] and took place at Center for Clinical Metabolic Research, Gentofte Hospital, University of Copenhagen, Denmark. In short, at baseline and after 1 year of treatment, participants met in the morning after a 10-h fast and had their waist and hip circumferences as well as blood pressure measured and underwent a 4 h 75 g-OGTT. Furthermore, participants underwent several examinations as described below. After baseline examinations, the women were randomized in blocks of varying sizes in a 1:1 ratio to treatment with either liraglutide 1.8 mg once-daily (initiated at 0.6 mg once-daily and escalated in steps of 0.6 mg per week) or placebo for 1 year. Placebo was identical in composition to liraglutide with the exception that it did not contain the active pharmaceutical ingredient. Novo Nordisk A/S who supplied the investigational drug, also provided the randomization list, which was transferred directly to an external company (Defactum, Denmark) who inserted it into the eCRF. The participants were stratified based on normal vs. prediabetes (impaired fasting glucose and/or impaired glucose tolerance) status. 

#### 2.3.1. Transient Elastography and Ultrasonography

At baseline and after 1 year of intervention, participants underwent transient elastography scanning (FibroScan^®^; Echosens, Paris, France) with assessment of intrahepatic fat by CAP (L.V. or S.F.) to evaluate liver stiffness due to fibrosis and steatosis, respectively. All liver stiffness results with an interquartile range (IQR) of less than 30% of the median value and a success rate of at least 60% were analyzed. CAP was analyzed when at least 10 successful acquisitions on the same patient were obtained. Steatosis severity was graded using CAP cutoffs by Eddowes et al.: S0 (<5% steatosis) for CAP < 302 dB/m, S1 (5–33% steatosis) for CAP 303–330 dB/m, S2 (34–65% steatosis) for CAP 331–336 dB/m, and S3 (>66% steatosis) for CAP > 337 dB/m [28]. At baseline and after 1 year, participants also underwent a real-time B-mode ultrasonography with a high-end ultrasound scanner (Logiq E9; General Electric, Milwaukee, WI, USA), using a convex probe (2.5–6 MHz), to evaluate hepatic steatosis. All examinations were performed by the same specialized radiologist (C.S.) who was blinded for the intervention. Steatosis was graded into mild (mild increase in hepatic echogenicity and normal visualization of diaphragm and intrahepatic vessel borders), moderate (moderate increase in hepatic echogenicity and slightly impaired visualization of diaphragm and intrahepatic vessel borders), and severe steatosis (marked increase in hepatic echogenicity, poor penetration of the posterior segment of the right liver lobe, and poor or no visualization of diaphragm and intrahepatic vessel borders), although we did not distinguish between the grades in the statistical analyses. 

#### 2.3.2. Dual Energy X-ray Absorptiometry

At baseline and after 1 year, participants were scanned in a DXA scanner (Lunar iDXA, GE Healthcare, Madison, WI, USA), and the accompanying software Encore version 16 was used to analyze data. Visceral fat mass was computed by subtracting subcutaneous fat mass from total abdominal fat mass in the predefined region. Fat mass in percent was calculated as total amount of fat divided by the total body weight (fat mass, bone mass, and lean body mass) multiplied by 100%.

#### 2.3.3. Alcohol Consumption

At baseline and after 1 year, the women filled in a questionnaire about alcohol consumption. We defined excessive alcohol intake as ≥20 g/day. None of the participants reported excessive alcohol intake on either of the assessment days. 

#### 2.3.4. Biochemical Measurements

For bedside measurement of plasma glucose, blood was distributed into fluoride tubes and centrifuged at room temperature immediately for 2 min at 7400 g. Plasma glucose concentrations were measured by the glucose oxidase method, using a glucose analyzer (model 2300 STAT Plus Analyzer; YSI, Yellow Springs, OH, USA). Plasma glucagon concentrations were measured by RIA with a C-terminal–specific glucagon antibody (no. 4305, University of Copenhagen, Denmark). The sensitivity of this RIA is approximately 1 pmol/L and the intraassay coefficient of variance (CV) is <6% [29]. Serum insulin concentrations were measured using two-sided electrochemiluminescence immunoassay (Siemens Healthcare, Ballerup, Denmark). The intraassay CV for this analysis is <2.7%. The serum concentrations of sCD163 and sMR were analyzed in duplicate using an in-house sandwich enzyme-linked immunosorbent assay on a BEP2000 ELISA analyzer (Dade Behring, Marburg, Germany) using monoclonal anti-CD163 (GHI/61, BD Pharmingen, San Jose, CA, USA) and biotinylated monoclonal anti-MR antibody (AM05589PU-S, Acris Antibodies, Rockville, MD, USA). For the sCD163 assay, the detection limit is below 6.25 µg/L and the with-in subject CV is 9.0% [30]. For the sMR assay, the mean was 0.23 µg/L and the 95% CI was 0.13–0.33 µg/L [31]. Liver function was assessed by ALT, AST, and GGT and like plasma cholesterol (total cholesterol and HDL, LDL, and VLDL cholesterol) and triglyceride concentrations, they were measured by standard methods at the Department of Clinical Biochemistry at Gentofte Hospital, University of Copenhagen, Hellerup, Denmark.

#### 2.3.5. Calculation of Indices

Insulin resistance was calculated using the HOMA2 calculator v2.2.3 (www.dtu.ox.ac.uk). The probability score of steatosis was calculated according to the validated FLI: (e^0.953×loge (triglycerides) + 0.139×BMI + 0.718×loge (ggt) + 0.053×waist circumference - 15.745^)/(1 + e^0.953×loge (triglycerides) + 0.139×BMI + 0.718×loge (ggt) + 0.053×waist circumference - 15.745^) × 100. This categorized the women into three groups: group 1 with FLI ≤ 30 (very low risk of steatosis), group 2 with FLI > 30 and <60 (intermediate risk of steatosis), and group 3 with FLI ≥ 60 (high risk of steatosis).

### 2.4. Statistical Methods 

The primary outcome measure (total area under the glucose curve) was used in the sample size calculation. With expected end-of-treatment values of 1713 mmol/L × min (standard deviation (SD) 212 mmol/L) and 1853 mmol/L × min (SD 212) in the intervention and placebo groups (raw data from [32]), respectively, and with α set to 5% and power to 90%, the estimated sample size was 98 participants (49 participants in each arm). The statistical analyses were performed using R version 3.3.3 “Another Canoe” (The R Foundation for Statistical Computing, Vienna, Austria) and GraphPad Prism version 7.02 software (GraphPad Software, San Diego, CA, USA) and carried out on all 82 participants who had an ultrasound scan at baseline and year one. Baseline data are expressed as mean with 95% CI except for the FLI and alcohol intake for which the results are expressed as median with IQR as these parameters were not normally distributed before or after log-transformation. Results of the intervention are expressed as delta mean with 95% CI for normally distributed data. Data were log-transformed when non-normally distributed and expressed as percentage change from baseline. The effect of liraglutide on development or resolution of NAFLD was examined with the chi square test. Baseline values between two groups were compared using unpaired *t*-test with Welch’s correction, which does not assume equal standard deviations for normally distributed data and Mann–Whitney’s U test for non-normally distributed data. Baseline comparisons between more than two groups were analyzed using ANOVA and Kruskal–Wallis test. Changes from baseline (repeated measures) were evaluated using linear mixed modelling with the “nlme” package in R, with a top-down modelling strategy. Participant number was chosen as random factor and a covariance structure based on NAFLD status was chosen and included according to log-likelihood ratios. Chi square test was used to evaluate appearance and disappearance of NAFLD between the treatment arms. *p*-values < 0.05 were considered significant.

## 3. Results

The present manuscript includes analyses of the population of women who attended abdominal ultrasound and transient elastography with CAP at baseline and after 1 year’s intervention. At baseline, participant characteristics were similar in the liraglutide and placebo groups (Table 1). We have previously published data from the original cohort [10,33,34].

### 3.1. Effects of the Intervention

#### 3.1.1. Abdominal Ultrasound, Transient Elastography with CAP, Liver Enzymes, and Inflammation Markers 

**Figure 1:**
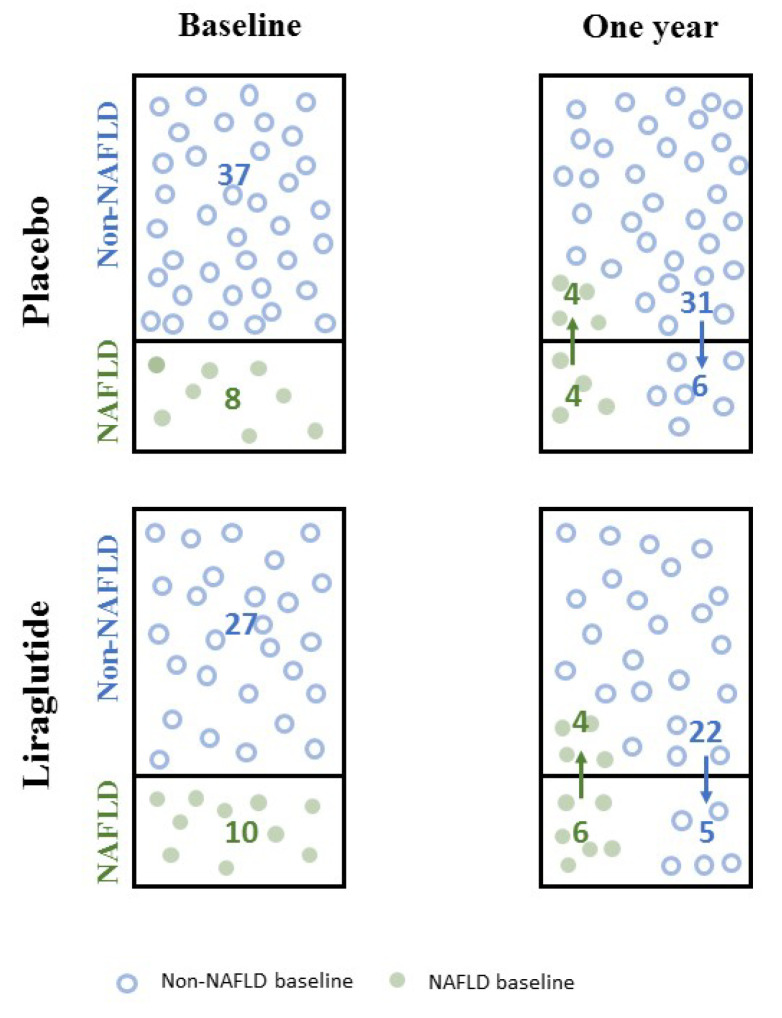
How many changed and how many remained in their nonalcoholic fatty liver disease (NAFLD)/non-NAFLD group after 1 year? In the placebo group, 4 of the 8 women with NAFLD at baseline were without NAFLD after 1 year and 6 of the 37 women without NAFLD at baseline developed NAFLD after 1 year. In the liraglutide group, 4 of the 10 women with NAFLD at baseline were without NAFLD after 1 year and 5 of the 27 women without NAFLD at baseline developed NAFLD after 1 year. (Section 3.1).

The abdominal ultrasound showed NAFLD in 18 women (22%) at baseline; of these, 10 (56%) were treated with liraglutide for 1 year. After 1 year, 4 (40%) of the 10 liraglutide-treated women with NAFLD at baseline and 4 (50%) of the placebo-treated women with NAFLD at baseline, no longer had ultrasonic evidence of steatosis. Five (19%) participants in the liraglutide group and six (16%) participants in the placebo group developed ultrasonically verified NAFLD during the intervention period (Figure 1). 

Chi square test showed that liraglutide had no effect on the appearance or disappearance of NAFLD (*p* = 0.74). Based on the TE, none of the included participants had significant fibrosis neither at baseline nor after 1 year. The liraglutide-treated group had a reduction in intrahepatic fat content as measured by CAP after 1 year (−28 (−44;−11) dB/m) compared to placebo (2 (−13;18) dB/m), this change was significant (*p* < 0.01). FLI was unchanged in both groups. Delta values of ALT and AST were similar in the two groups (Table 1). The inflammation marker sCD163 was unchanged in both groups. sMR delta values were not significantly different between the groups.

#### 3.1.2. Body Weight and Body Composition

The liraglutide group had larger mean reductions in body weight and BMI (body weight (mean (95% CI)): −4.7 (−6.4;−2.9) kg; BMI: −1.7 (−2.3;−1.1) kg/m^2^) when compared to the placebo group (body weight: −1.4 (−3.0;0.3) kg; BMI: −0.5 (−1.1;0.1) kg/m^2^) (body weight: *p <* 0.01; BMI: *p <* 0.01) (Table 1). Changes in total fat mass, visceral fat mass and android-to-gynoid fat mass ratio did not differ between the liraglutide and the placebo groups. Changes in waist circumference and waist-hip ratio were similar in the two groups.

#### 3.1.3. Plasma Glucose, HbA1c, Insulin, Glucagon, and Insulin Resistance

As opposed to placebo, liraglutide reduced fasting plasma glucose (−0.4 (−0.6;−0.3) mmol/L vs. −0.0 (−0.2;0.1); *p* < 0.01) and HbA1c (−2.0 (−3.3;−0.6) mmol/mol (−0.18 (−0.30;−0.06)%) vs. 0.6 (−0.4;1.5) mmol/mol (0.05 (−0.03;−0.13)%); *p* < 0.01) (Table 1). No changes were observed in fasting insulin, fasting glucagon, or HOMA-IR in either of the groups (Table 1).

#### 3.1.4. Heart Rate, Blood Pressure, and Lipids

Liraglutide caused an increase in heart rate (5.5 (2.2;8.9) bpm) as opposed to a decrease in the placebo group (−2.7 (−5.2;−0.2) bpm) (*p* < 0.01). Delta values of systolic and diastolic blood pressure were comparable in the two groups. Total cholesterol, HDL cholesterol, VLDL cholesterol, and triglycerides delta values were similar in both groups (Table 1).

### 3.2. Effect of Liraglutide in Participants with and without NAFLD 

We investigated if there was an effect of liraglutide in women with pGDM and NAFLD compared to placebo and if the effect of liraglutide was different in women with pGDM and NAFLD compared to women with pGDM without NAFLD. Details of baseline characteristics are given in Table S1.

#### TE with CAP, Liver Enzymes, and Inflammation Markers

CAP-value changes from baseline to year one for the four subgroups were comparable (liraglutide non-NAFLD vs. NAFLD: *p* = 0.44 and placebo non-NAFLD vs. NAFLD: *p* = 0.86). Neither liraglutide nor placebo changed FLI regardless of presence or absence of NAFLD (Table 2). Delta values for GGT and AST were similar in the liraglutide and placebo subgroups. The observed increase in ALT in the placebo group was confined to the subgroup without NAFLD (*p* < 0.05). ALT did not change in the liraglutide subgroups (*p* = 0.81). Compared to the liraglutide non-NAFLD group, the inflammation marker sCD163 decreased significantly in the liraglutide NAFLD group (*p* < 0.01) but was unaltered in the placebo subgroups. Delta values of sMR were similar in the liraglutide and placebo subgroups (Table 2). Additional parameters for the subgroup analyses are available in Table S2.

## 4. Discussion

In this study, liraglutide had no effect on the proportion of patients with ultrasonically verified NAFLD in women with pGDM. Compared to placebo, however, liraglutide reduced intrahepatic fat as measured by CAP as well as body weight, HbA1c, and fasting plasma glucose. 

The quantification of hepatic steatosis is of growing clinical relevance because increasing steatosis may favor progression of fibrosis and because it may be used for estimating the therapeutic success of interventions. Liver biopsy is the gold standard for assessing NAFLD/NASH, and by using histology, the degree of hepatic steatosis can be assessed. The CAP, which is included in the TE, evaluates steatosis based on shear-wave propagation and its performance is supported by several biopsy-controlled clinical studies. Based on previously defined cutoff values for different grades of CAP-measured steatosis, liraglutide was associated with a reduction of approximately one steatosis grade from mild to absent [35,36]. The extent to which this will translate to clinically relevant outcomes (e.g., NASH or cirrhosis) is uncertain. However, it is notable that liraglutide has a significant beneficial effect even in patients with mild NAFLD [35,36]. Ultrasound does not reliably detect steatosis below 20% or when the BMI is above 40 kg/m^2^ and the method is, to some extent, operator-dependent. Most of the participants in this study had BMI below 40 kg/m^2^. Nonetheless, ultrasound scanning was too coarse method to detect the small improvement in steatosis, which was induced by liraglutide. The changes were, however, detectable using CAP which, in addition, allows quantification of hepatic fat and thus, seems to be the better choice in future studies.

Several studies have investigated the effects of GLP-1RAs in patients with type 2 diabetes and NAFLD and with only a few exceptions [18,20,37,38] they all found that GLP-1RA treatment was able to improve NAFLD either by reducing intrahepatic fat, visceral fat, or fibrosis [12,13,14,15,16,22,23,24,25]. In addition, a study of patients with NASH showed that 48 weeks of liraglutide treatment led to resolution of NASH in 39% of the patients compared with 2% in the placebo group [11]. In addition, two studies in women with polycystic ovary syndrome found that liraglutide reduced the serum fibrosis marker procollagen type 3 amino-terminal peptide, visceral adipose tissue, and liver fat content [17,39]. The present study included participants without significant fibrosis based on the TE. None of the participants underwent a liver biopsy, but it is likely that most women had simple steatosis, due to their low liver enzyme levels and lack of symptoms. In agreement with previous studies, we found reduced liver fat in the liraglutide-treated group. Liraglutide reduced the BMI but did not decrease the visceral fat mass nor the proportion of patients with ultrasonically verified NAFLD. Only one of the previous studies which investigated the effects of GLP-1RAs in patients with type 2 diabetes and NAFLD used CAP to measure steatosis. In that trial, 12 weeks’ treatment of type 2 diabetes patients with dulaglutide did not reduce CAP, possibly due to too short intervention period [23]. Our cohort of women was relatively young and healthy compared to the patients with type 2 diabetes who were included in many of the previous trials which may explain why the reduction of liver fat can only be detected with CAP but not by ultrasound scanning. The women participating in the present study had liver enzyme levels within the normal range but, nonetheless, we found unaltered ALT levels in the liraglutide group as opposed to an increase in the placebo group and unchanged GGT levels. This is in accordance with other studies using GLP-1RAs showing either unaltered or improvements of elevated liver enzyme levels [13,15,16,20,22,23]. 

Both preclinical and clinical data on a number of markers suggest anti-inflammatory effects of GLP-1RAs [40,41]. Specifically, a study in obese patients with dysregulated type 2 diabetes found that 8 weeks of intervention with liraglutide 1.2 mg/daily significantly reduced sCD163 levels [19]. In contrast, we found no effect of liraglutide on sCD163 and sMR. As of yet, associations of sMR in obesity and diabetes have not been described, whereas sCD163 levels are increased in both obesity and type 2 diabetes and, hence, given that our cohort is both leaner and nondiabetic it may explain why our intervention with liraglutide does not reduce sCD163 levels. In addition, it suggests that liraglutide’s reduction of hepatic fat is not due to reduced inflammation, however, this can only be ascertained histologically. In congruence with previous trials using liraglutide, we found that compared to placebo, liraglutide reduced body weight, BMI, fasting plasma glucose, and HbA1c [21]. Both body weight reductions of only 3 kg [42] and improvements in glycemia [38] have, on their own, been demonstrated to reduce hepatic steatosis. Although previous in vitro studies have suggested that GLP-1RAs suppress hepatic lipogenesis and activate hepatic fatty acid beta-oxidation, this has not been confirmed in humans [43]. In the current study, we are not able to assess if the improvement in steatosis was attributable to body weight reductions and/or improved glycemia.

When evaluating the treatment arms according to presence and absence of steatosis, the liraglutide-treated group with NAFLD experienced decreased sCD163 levels, however, this difference was not significantly different from that of the placebo NAFLD group, suggesting that liraglutide has no effect on this parameter in women with pGDM and NAFLD per se. The decreased sCD163 was in congruence with sCD163 being linked to obesity and hence decreasing after body weight loss [21]. 

This study was limited by the use of ultrasound to detect steatosis as ultrasound does not reliably detect steatosis below 20% or when the BMI is above 40 kg/m^2^, and it is operator dependent [3]. We have accommodated for the latter by ensuring that the same (blinded for the therapies) specialized radiologist (C.S.) performed and described all examinations. Despite this mitigation, the FLI calculations in the subgroups indicate that there are women with steatosis in the non-NAFLD subgroups as their average FLI is close to the lower steatosis cutoff value of 60 and hence, the women in the non-NAFLD groups resemble those in the NAFLD groups. This could be a contributing factor to the similarities of other parameters between the subgroups. Liver biopsy remains the gold standard for determining amount of liver fat and is the only method for distinguishing NAFLD from NASH. However, biopsy is an invasive procedure and for that reason, it was judged unethical in this generally healthy cohort. Application of ^1^H-MRI would have allowed noninvasive quantification of liver fat but this is time consuming and an expensive machinery with often limited accessibility. The randomized, double-blinded, placebo-controlled design of the study is an obvious strength. However, the study was powered for the primary outcome, which was the change in glucose tolerance from baseline to week 52 as measured by AUC for the PG excursion following a 4 h 75 g-OGTT, and not for determining effects of the intervention in the NAFLD and non-NAFLD subgroups. The resulting small number of participants in each group, particularly the placebo NAFLD group (*n* = 8) and the liraglutide NAFLD group (*n* = 10), increases the risk of type 2 errors and the results can therefore only be considered exploratory.

## 5. Conclusions

In conclusion, 1 year’s liraglutide treatment significantly reduced intrahepatic fat assessed by CAP, but an effect on the presence of NAFLD could not be detected in these women. In addition, liraglutide treatment of overweight or obese, nondiabetic women with pGDM reduced body weight, fasting plasma glucose, and HbA1c compared to placebo treatment. The study is ongoing with a 4-year open-label extension, which has the potential to unveil the long-term effects of liraglutide on women with pGDM and NAFLD.

## Figures and Tables

**Table 1:** Baseline characteristics and change from baseline to year one.

	Placebo (A)	Liraglutide (B)	*p-*Value A vs. B	Placebo (C)	Liraglutide (D)	*p-*Value C vs. D
Number (*n*)	45	37		45	37	
Age (years)	38.3 (35.5;41.2)	38.8 (34.3;40.7)	0.42	–	–	–
Body weight (kg)	83.9 (76.3;92.6)	89.0 (75.4;107.2)	0.14	−1.4 (−3.0;0.3)	**−4.7 (−6.4;−2.9)**	**<0.01**
Body mass index (kg/m^2^)	30.6 (28.4;33.0)	32.1 (27.4;36.3)	0.10	−0.5 (−1.1;0.1)	**−1.7 (−2.3;−1.1)**	**<0.01**
Waist circumference (cm)	104 (101;106)	104 (99;110)	0.78	−2.2 (−4.5;0.1)	**−3.4 (−6.0;−0.9)**	0.48
Waist:hip ratio	0.9 (0.9;0.9)	0.9 (0.9;0.9)	0.75	0.00 (−0.02;0.03)	−0.01 (−0.04;0.02)	0.57
Systolic blood pressure (mmHg)	128 (125;132)	125 (121;129)	0.19	**−6.9 (−10.1;−3.7)**	**−6.4 (−10.0;−2.9)**	0.84
Diastolic blood pressure (mmHg)	82 (79;84)	80 (78;83)	0.49	**−8.0 (−10.3;−5.6)**	**−5.1 (−7.7;−2.5)**	0.11
Heart rate (beats/min)	68 (64;75)	69 (62;78)	0.66	**−2.7 (−5.2;−0.2)**	**5.5 (2.2;8.9)**	<0.01
Hemoglobin A1c (mmol/mol)	31.8 (28.6;34.3)	33.0 (29.5;36.0)	0.38	0.6 (−0.4;1.5)	**−2.0 (−3.3;−0.6)**	<0.01
Hemoglobin A1c (%)	5.1 (5.0;5.2)	5.1 (5.0;5.3)	0.38	0.05 (−0.03;0.13)	**−0.18 (−0.30;−0.06)**	<0.01
Parity (n)	2.1 (1.9;2.3)	2.3 (2.0;2.6)	0.27	–	–	–
Time since first GDM pregnancy (years)	4.9 (4.2;5.7)	5.2 (4.1;6.2)	0.69	–	–	–
Total cholesterol (mmol/L)	4.6 (4.4;5.4)	5.1 (4.4;5.7)	0.15	**0.95 (0.91;1.00)**	**0.93 (0.89;0.97)**	0.49
HDL (mmol/L)	1.2 (121;1.3)	1.2 (1.1;1.3)	0.99	**−0.1 (−0.2;0.0)**	**−0.1 (−0.1;0.0)**	0.35
LDL (mmol/L)	2.9 (2.4;3.5)	3.3 (2.4;3.7)	0.25	−0.1 (−0.3;0.1)	**−0.2 (−0.4;0.0)**	0.51
VLDL (mmol/L)	0.5 (0.3;0.6)	0.5 (0.4;0.7)	0.31	0.97 (0.86;1.09)	0.92 (0.80;1.06)	0.56
Triglycerides (mmol/L)	1.0 (0.9;1.2)	1.2 (1.0;1.4)	0.31	0.94 (0.84;1.05)	0.90 (0.78;1.02)	0.53
Android:gynoid fat ratio	1.1 (1.0;1.2)	1.1 (1.0;1.2)	0.99	**−0.03 (−0.05;−0.01)**	−0.01 (−0.03;0.00)	0.81
Total fat mass (%)	43.7 (40.4;47.0)	45.4 (39.3;50.0)	0.41	**−1.2 (−2.1;−0.3)**	**−2.0 (−2.9;−1.1)**	0.23
Visceral fat mass (g)	918 (785;1074)	977 (778;1225)	0.66	**0.89 (0.80;0.99)**	**0.84 (0.76;0.92)**	0.41
GGT (U/L)	17.5 (15.7;19.6)	19.2 (16.3;22.5)	0.36	1.4 (−0.4;3.3)	**1.7 (0.6;2.7)**	0.84
ALT (U/L)	24.6 (20.3;29.0)	24.7 (22.2;27.1)	0.98	**1.17 (1.06;1.31)**	0.99 (0.89;1.00)	**0.05**
AST (U/L)	26.5 (24.0;28.9)	26.4 (24.5;28.2)	0.94	0.99 (0.91;1.06)	0.93 (0.85;1.07)	0.27
FLI	61.7 (36.0;86.6)	69.8 (33.5;80.2)	0.66	−4.2 (−17.3;5.8) *p =* 0.43	−4.0 (−13.0;0.8) *p =* 0.37	0.52
HOMA_IR_	1.7 (1.4;1.9)	2.0 (1.7;2.3)	0.06	−0.1 (−0.2;0.1)	0.0 (−0.2;0.2)	0.36
Transient elastography (kPa)	4.1 (3.8;4.4)	4.5 (3.9;5.0)	0.29	–	–	–
CAP (db/m)	269 (246;284)	276 (251;303)	0.46	2.3 (−13.1;17.6)	**−28.0 (−44.6;−11.4)**	<0.01
S0 (n)	37	28	–	–	–	–
S1 (n)	4	5	–	–	–	–
S2 (n)	0	1	–	–	–	–
S3 (n)	4	3	–	–	–	–
sCD163 (mg/L)	1.6 (1.5;1.8)	1.6 (1.5;1.8)	0.82	0.04 (−0.09;0.17)	−0.04 (−0.14;0.06)	0.62
sMR (mg/L)	0.22 (0.20;0.24)	0.22 (0.20;0.24)	0.89	**0.03 (0.01;0.05)**	0.01 (−0.01;0.03)	0.34
Fasting plasma glucose (mmol/L)	5.3 (5.2;5.5)	5.5 (5.3;5.6)	0.19	0.0 (−0.2;0.1)	**−0.4 (−0.6;−0.3)**	**<0.01**
Fasting serum insulin (pmol/L)	82.8 (57.4;109.8)	101.7 (69.4;134.9)	0.07	−3.5 (−11.5;4.5)	3.7 (−8.3;15.7)	0.23
Fasting plasma glucagon (pmol/L)	5.8 (5.1;6.6)	6.5 (5.9;7.3)	0.15	0.5 (−0.2;1.2)	0.4 (−0.6;1.5)	0.87
Alcohol (units/week)	1.0 (1.0;1.0)	1.0 (0.5;2.0)	0.91	0.0 (−0.5;0.9) *p =* 0.18	0.0 (−0.8;1.0) *p =* 0.36	0.94

Normally distributed data are mean and (95% CI) and if zero is not included in the 95% CI, the change is significant (marked in bold). Log transformed data are ratio and (95% CI) and if “1” is not included in the 95% CI, the change is significant (marked in bold). Alcohol and FLI were non-normally distributed and hence shown as median with IQR. Normally distributed data (if necessary, after log-transformation) were analyzed with unpaired *t*-test with Welch’s correction and non-normally distributed data were analyzed with Mann–Whitney’s U test. **A** and **B**: baseline data. Baseline log-transformed parameters: fasting plasma glucose, visceral fat mass, HDL, triglycerides, GGT, and fasting plasma glucagon. **C** and **D**: change from baseline. Delta log-transformed parameters: ALT, AST, total cholesterol, triglycerides, VLDL, and visceral fat mass. ALT, alanine aminotransferase; AST, aspartate aminotransferase; CAP, continuous attenuation parameter; FLI, fatty liver index; GGT, gamma glutamyltransferase; S0, steatosis grade 0 (≤5% fat infiltrated hepatocytes); S1, steatosis grade 1 (6–33% fat infiltrated hepatocytes); S2, steatosis grade 2 (34–66% fat infiltrated hepatocytes); S3, steatosis grade 3 (≥67% fat infiltrated hepatocytes); sCD163, soluble CD163; sMR, soluble mannose receptor.

**Table 2:** Changes from baseline to year one for subgroups with and without nonalcoholic fatty liver disease (NAFLD) at baseline.

	Placebo		Liraglutide		*p*-Value	*p-*Value	*p-*Value
	Non-NAFLD (C1)	NAFLD (C2)	Non-NAFLD (D1)	NAFLD (D2)	C1 vs. C2	D1 vs. D2	C2 vs. D2
**Number (*n*)**	37	8	27	10			
**CAP (db/m)**	2.9 (−13.5;19.2)	−0.6 (−53.5;52.2)	−32.0 (−52.2;−11.8)	−17.3 (−45.0;10.4)	0.86	0.44	0.54
**FLI**	−4.4 (−19.0;5.5) *p =* 0.45	0.4 (−13.2;5.3) *p =* 1.0	−8.1 (−13.8;−0.1) *p =* 0.17	−1.2 (−1.9;2.9) *p =* 0.97	1.0	0.13	0.96
**GGT (U/L)**	1.9 (−0.2;4.0)	−0.6 (−6.1;4.8)	1.2 (−0.1;2.5)	**3.0 (1.2;4.8)**	0.31	0.12	0.12
**ALT (U/L)**	1.2 (1.1;1.3)	0.8 (0.5;1.3)	1.0 (0.9;1.1)	1.0 (0.7;1.3)	**<0.05**	0.81	0.40
**AST (U/L)**	1.0 (0.9;1.1)	0.9 (0.7;1.2)	0.9 (0.9;1.0)	0.9 (0.7;1.0)	0.25	0.26	0.92
**sCD163 (mg/L)**	0.0 (−0.1;0.2)	−0.2 (−1.0;0.6)	0.0 (−0.1;0.1)	**−0.3 (−0.5;−0.1)**	0.45	**<0.01**	0.73
**sMR (mg/L)**	0.02 (0.00;0.05)	0.05 (−0.03;0.14)	0.01 (−0.01;0.03)	0.01 (−0.01;0.08)	0.34	0.59	0.60

The liraglutide- and placebo-treated groups were subdivided according to their NAFLD-status at baseline. Data are changes from baseline to year one. Normally distributed data are mean and (95% CI) and if zero is not included in the 95% CI, the change is significant (marked in bold). Log transformed data are ratio and (95% CI), and if “1” is not included in the 95% CI, the change is significant (marked in bold). Normally distributed data (if necessary, after log-transformation) are analyzed with unpaired *t*-test with Welch’s correction. Log-transformed parameters: ALT and AST. FLI is median with IQR. These data are non-normally distributed and are analyzed using Mann–Whitney’s U test. ALT, alanine aminotransferase; AST, aspartate aminotransferase; CAP, continuous attenuation parameter; GGT, gamma-glutamyltransferase; sCD163, soluble CD163; sMR, soluble mannose receptor.

## Supplementary Materials

The following are available online at https://www.mdpi.com/2077-0383/9/10/3213/s1, Table S1: Baseline values in nonalcoholic fatty liver disease (NAFLD) subgroups; Table S2: Changes from baseline to year one for subgroups with and without NAFLD.
