# Supplementary material for: One Year’s Treatment with the Glucagon-Like Peptide 1 Receptor Agonist Liraglutide Decreases Hepatic Fat Content in Women with Nonalcoholic Fatty Liver Disease and Prior Gestational Diabetes Mellitus in a Randomized, Placebo-Controlled Trial
Source: J Clin Med. 2020 Oct 6;9(10):3213. doi: 10.3390/jcm9103213 (PMC7601647; doi:10.3390/jcm9103213)
Supplement: Supplementary file 1 [file jcm-09-03213-s001.pdf]

Table S1. Baseline values in NAFLD subgroups. Data are mean with 95% CI.

	Placebo		Liraglutide		*p*-value	*p*-value	*p*- value
	Non-NAFLD (A1)	NAFLD (A2)	Non-NAFLD (B1)	NAFLD (B2)	A1 vs. A2	B1 vs. B2	A2 vs. B2
**Number (n)**	37	8	27	10			
**Age (years)**	38.8 [37.5;40.2]	37.7 [32.9;41.2]	38.5 [36.5;40.5]	36.0 [32.9;39.1]	0.53	0.13	0.41
**Body weight (kg)**	84.9 [80.9;88.9]	90.3 [90.7;97.4]	86.3 [79.1;93.6]	105.6 [90.7;120.5]	0.37	**0.001**	**0.04**
**Body mass index (kg/m²)**	30.6 [29.5;31.7]	32.1 [32.6;34.8]	31.3 [29.1;33.4]	36.3 [32.6;40.0]	0.36	**0.002**	**0.04**
**Waist circumference (cm)**	103.2 [100.0;106.3]	105.3 [108.1;112.7]	99.3 [93.5;105.2]	118.2 [108.1;128.3]	0.66	**<0.0001**	**0.03**
**Waist:hip ratio**	0.9 [0.9;0.9]	0.9 [0.9;1.0]	0.9 [0.8;0.9]	1.0 [0.9;1.0]	0.87	**0.0003**	0.14
**Systolic blood pressure (mmHg)**	129 [125;132]	128 [118;137]	125 [121;129]	125 [117;135]	0.84	0.92	0.59
**Diastolic blood pressure (mmHg)**	82 [79;84]	82 [72;92]	79 [76;83]	83 [78;88]	0.95	0.24	0.76
**Heart rate (beats/min)**	67.7 [65.1;70.2]	74.6 [65.3;79.3]	69.2 [64.9;73.5]	71.6 [65.3;77.9]	**0.05**	0.46	0.47
**Hemoglobin A1c (mmol/mol)**	31.6 [30.3;32.9]	33.2 [31.1;37.0]	32.1 [30.6;33.7]	34.1 [31.1;37.0]	0.29	0.20	0.67
**Hemoglobin A1c (%)**	5.0 [4.9;5.2]	5.2 [4.8;5.5]	5.1 [4.9;5.2]	5.3 [5.0;5.5]	0.29	0.20	0.66
**Total cholesterol (mmol/L)**	4.7 [4.4;5.0]	5.0 [4.5;5.6]	4.9 [4.6;5.2]	5.3 [4.5;6.1]	0.37	0.21	0.43
**HDL (mmol/L)**	1.3 [1.2;1.3]	1.2 [1.0;1.4]	1.3 [1.2;1.4]	1.2 [1.0;1.4]	0.34	0.24	0.98
**LDL**	2.9 [2.7;3.2]	3.2 [2.8;3.6]	3.1 [2.8;3.4]	3.4 [2.8;3.9]	0.35	0.27	0.59
**VLDL (mmol/L)**	0.5 [0.4;0.6]	0.6 [0.4;0.9]	0.5 [0.4;0.7]	0.7 [0.4;1.0]	0.43	0.12	0.36
**Triglycerides (mmol/L)**	1.0 [0.9;1.2]	1.2 [0.7;1.9]	1.1 [0.9;1.3]	1.4 [1.0;2.1]	0.47	0.11	0.35
**Android:gynoid fat ratio**	1.1 [1.0;1.1]	1.2 [1.1;1.4]	1.1 [1.0;1.1]	1.1 [1.1;1.2]	**0.05**	0.27	0.48
**Total fat mass (%)**	43.2 [41.9;44.6]	45.6 [46.8;48.9]	43.0 [40.2;45.8]	49.4 [46.8;52.0]	0.25	**0.002**	0.13
**Visceral fat mass (g)**	863 [724;1030]	1222 [855;1750]	822 [627;1076]	1556 [1143;2113]	0.12	**0.003**	0.37
**GGT (U/L)**	17.3 [15.2;19.6]	19.0 [14.7;24.5]	19.6 [15.9;24.2]	18.2 [14.3;23.1]	0.57	0.64	0.84
**ALT (U/L)**	22.6 [19.6;25.6]	34.1 [20.9;57.5]	24.4 [21.3;27.5]	27.7 [20.9;29.9]	**0.01**	0.82	0.11
**AST (U/L)**	25.7 [23.1;28.2]	30.3 [22.6;38.2]	25.9 [23.9;27.8]	27.8 [22.6;32.8]	0.09	0.47	0.44
**FLI**	55.8 [47.9;63.6]	67.5 [73.4;84.5]	52.4 [41.0;63.8]	83.3 [73.4;93.3]	0.27	**0.002**	0.16
**CAP (db/m)**	257 [243;270]	325 [280;370]	269 [248;290]	305 [268;342]	**0.0004**	0.06	0.39
**sCD163 (mg/L)**	1.5 [1.4;1.6]	2.3 [1.6;3.0]	1.6 [1.4;1.8]	2.0 [1.6;2.3]	**0.0001**	**0.05**	0.17
**sMR (mg/L)**	0.2 [0.2;0.2]	0.3 [0.2;0.3]	0.2 [0.2;0.2]	0.2 [0.2;0.3]	**0.03**	0.79	0.17

**$HOMA_{IR}$**	1.5 [1.3;1.7]	2.4 [1.9;3.2]	1.8 [1.5;2.1]	2.6 [1.9;3.4]	**0.01**	**0.003**	0.43
**Fasting plasma glucose (mmol/L)**	5.4 [5.2;5.5]	5.3 [4.8;5.9]	5.5 [5.3;5.7]	5.5 [5.1;5.9]	0.83	0.97	0.44
**Fasting serum insulin (pmol/L)**	79.8 [69.4;90.3]	126.5 [98.1;171.2]	94.4 [78.4;110.4]	141.9 [98.1;185.7]	**0.005**	**0.002**	0.43
**Fasting plasma glucagon (pmol/L)**	5.4 [4.7;6.2]	7.9 [5.9;10.6]	6.4 [5.6;7.4]	6.8 [5.8;8.1]	**0.01**	0.65	0.42
**Alcohol (units/week)**	1.7 [1.0;2.3]	0.6 [0.0;1.2]	1.3 [0.8;1.7]	1.1 [0.2;2.0]	0.05	0.53	0.37

Normally distributed data and data that were normally distributed after log transformation (fasting plasma glucose, visceral fat mass, HDL, triglycerides, GGT and fasting glucagon) were analyzed with one-way ANOVA. Data that were not normally distributed after log transformation (FLI and alcohol intake) were analyzed using the non-parametric Kruskall-Wallis test with Dunn's multiple comparisons post hoc test. ALT, alanine aminotransferase; AST, aspartate aminotransferase; CAP, continuous attenuation parameter; FLI, fatty liver index; GGT, gamma glutamyl-transferase; $HOMA_{IR}$, homeostasis model assessment 2; sCD163, soluble CD163; sMR, soluble mannose receptor.

Table S2. Changes from baseline to year one for subgroups with and without NAFLD.

	Placebo		Liraglutide		*p* value	*p* value	*p* value
	**Non-NAFLD (C1)**	**NAFLD (C2)**	**Non-NAFLD (D1)**	**NAFLD (D2)**	**C1 vs. C2**	**D1 vs. D2**	**C2 vs. D2**
**Number (n)**	37	8	27	10			
**Body weight (kg)**	-1.4 [-3.1;0.3]	-1.2 [-7.4;5.0]	-4.8 [-6.8;-2.8]	-4.3 [-8.5;0.0]	0.92	0.78	0.34
**Body mass index (kg/m^2)**	-0.5 [-1.1;0.1]	-0.4 [-2.6;1.8]	-1.8 [-2.6;-1.1]	-1.5 [-2.8;-0.1]	0.92	0.63	0.35
**Waist circumference (cm)**	-2.6 [-5.3;0.0]	-0.4 [-5.5;4.8]	-3.6 [-6.7;-0.4]	-3.1 [-8.0;1.9]	0.46	0.86	0.40
**Waist:hip ratio**	0.0 [0.0;0.0]	0.0 [0.0;0.1]	0.0 [-0.1;0.0]	0.0 [0.0;0.1]	0.47	0.28	0.79
**Systolic blood pressure (mmHg)**	**-7.7 [-11.2;-4.2]**	-3.3 [-10.7;4.3]	-6.4 [-10.8;-1.9]	-6.5 [-13.8;0.9]	0.28	0.98	0.45
**Diastolic blood pressure (mmHg)**	**-8.0 [-10.3;-5.6]**	**-7.0 [-12.8;-1.1]**	**-4.3 [-7.3;-1.2]**	**-7.4 [-12.4;-2.4]**	0.71	0.28	0.92
**Heart rate (bpm)**	-2.3 [-5.3;0.7]	-4.6 [-11.1;2.0]	3.3 [-6.4;13.0]	**5.8 [1.3;10.3]**	0.41	0.10	0.08
**Hemoglobin A1c (mmol/mol)**	0.6 [-0.4;1.7]	0.1 [-2.0;2.3]	**-1.6 [-3.2;-0.1]**	-2.9 [-6.0;0.2]	0.67	0.41	0.10
**Hemoglobin A1c (%)**	0.06 [-0.04;0.16]	0.01 [-0.18;0.20]	-0.15 [-0.29;-0.01]	-0.26 [-0.55;0.02]	0.67	0.41	0.10
**Total cholesterol (mmol/L)**	**0.9 [0.9;1.0]**	1.0 [0.8;1.1]	**0.9 [0.9;1.0]**	**0.9 [0.9;1.0]**	0.73	0.75	0.50
**HDL (mmol/L)**	**-0.1 [-0.2;-0.1]**	-0.1 [-0.2;0.1]	-0.1 [-0.1;0.0]	-0.1 [-0.2;0.9]	0.42	0.90	0.97
**LDL**	-0.1[-0.3;0.1]	-0.2[-0.6;0.3]	-0.2[-0.4;0.1]	-0.3[-0.7;0.0]	0.81	0.40	0.57
**VLDL (mmol/L)**	0.9 [0.8;1.0]s	1.2 [0.8;1.8]	0.9 [0.8;1.1]	1.0 [0.7;1.4]	**0.05**	0.62	0.32
**Triglycerides (mmol/L)**	**0.9 [0.8;1.0]**	1.2 [0.8;1.7]	0.9 [0.7;1.0]	1.0 [0.7;1.3]	0.06	0.50	0.39
**Android:gynoid fat ratio**	**-0.03 [-0.05;-0.01]**	-0.01 [-0.07;0.04]	-0.03 [-0.07;-0.01]	-0.01 [-0.03;0.01]	0.53	0.56	0.92
**Fat mass (%)**	**-1.2 [-2.1;-0.2]**	-1.2 [-4.4;2.1]	**-2.3 [-3.4;-1.1]**	-1.1 [-2.5;0.4]	0.98	0.23	0.95
**Visceral fat (g)**	0.9 [0.8;1.0]	1.1 [0.8;1.4]	**0.8 [0.7;0.9]**	1.0 [0.8;1.1]	0.52	0.09	0.21
**$HOMA_{IR}$**	0.0 [-0.2;0.0]	-0.3 [-0.8;0.1]	0.1 [-0.2;0.3]	0.0 [-0.7;0.6]	0.24	0.76	0.40
**Fasting plasma glucose (mmol/L)**	0.0 [-0.2;0.1]	-0.1 [-0.4;0.2]	**-0.4 [-0.6;-0.3]**	-0.4 [-0.8;0.1]	0.61	0.56	0.31
**Fasting serum insulin (pmol/L)**	-2.4 [-10.9;6.1]	-17.9 [-43.1;7.3]	5.1 [-7.1;17.3]	0.0 [-35.0;34.9]	0.14	0.70	0.38
**Fasting plasma glucagon (pmol/L)**	0.5 [-0.2;1.3]	0.4 [-1.8;2.6]	0.0 [-1.1;1.1]	**3.5 [1.0;6.1]**	0.93	**0.003**	0.06

**Alcohol intake (units/week)**	0.0 (-0.5;0.5) *P* = 0.52	1.0 (0.0;1.0) *P* = 0.10	0.0 (0.0;1.0) *P* = 0.17	-0.9 (-1.1;-0.1) *P* = 1.0	0.41	0.08	0.09

Normally distributed data are mean and [95% CI] and if zero is not included in the 95% CI, the change is significant. Log transformed data are ratio and [95% CI] and if one is not included in the 95% CI, the change is significant. Normally distributed data (if necessary after log-transformation) are analyzed with unpaired t-test with Welch's correction. Log-transformed parameters: total cholesterol, triglycerides, VLDL and visceral fat mass. Alcohol intake is median with (IQR). These data are non-normally distributed and are analyzed using Mann-Whitney's U-test. $HOMA_{IR}$, homeostasis model assessment.
